# Local genetic covariance between serum urate and kidney function estimated with Bayesian multitrait models

**DOI:** 10.1093/g3journal/jkac158

**Published:** 2022-07-25

**Authors:** Alexa S Lupi, Nicholas A Sumpter, Megan P Leask, Justin O’Sullivan, Tayaza Fadason, Gustavo de los Campos, Tony R Merriman, Richard J Reynolds, Ana I Vazquez

**Affiliations:** Department of Epidemiology and Biostatistics, Michigan State University, East Lansing, MI 48824, USA; Institute for Quantitative Health Science and Engineering, Systems Biology, Michigan State University, East Lansing, MI 48824, USA; Department of Medicine, The University of Alabama at Birmingham, Birmingham, AL 35294, USA; Department of Medicine, The University of Alabama at Birmingham, Birmingham, AL 35294, USA; Department of Biochemistry, University of Otago, Dunedin 9016, New Zealand; Liggins Institute, The University of Auckland, Auckland 1142, New Zealand; Liggins Institute, The University of Auckland, Auckland 1142, New Zealand; Department of Epidemiology and Biostatistics, Michigan State University, East Lansing, MI 48824, USA; Institute for Quantitative Health Science and Engineering, Systems Biology, Michigan State University, East Lansing, MI 48824, USA; Department of Statistics and Probability, Michigan State University, East Lansing, MI 48824, USA; Department of Medicine, The University of Alabama at Birmingham, Birmingham, AL 35294, USA; Department of Medicine, The University of Alabama at Birmingham, Birmingham, AL 35294, USA; Department of Epidemiology and Biostatistics, Michigan State University, East Lansing, MI 48824, USA; Institute for Quantitative Health Science and Engineering, Systems Biology, Michigan State University, East Lansing, MI 48824, USA

**Keywords:** serum urate, serum creatinine, UK Biobank, local genetic covariance, eGFR, gout, hyperuricemia, chronic kidney disease, pleiotropy, multitrait

## Abstract

Hyperuricemia (serum urate >6.8 mg/dl) is associated with several cardiometabolic and renal diseases, such as gout and chronic kidney disease. Previous studies have examined the shared genetic basis of chronic kidney disease and hyperuricemia in humans either using single-variant tests or estimating whole-genome genetic correlations between the traits. Individual variants typically explain a small fraction of the genetic correlation between traits, thus the ability to map pleiotropic loci is lacking power for available sample sizes. Alternatively, whole-genome estimates of genetic correlation indicate a moderate correlation between these traits. While useful to explain the comorbidity of these traits, whole-genome genetic correlation estimates do not shed light on what regions may be implicated in the shared genetic basis of traits. Therefore, to fill the gap between these two approaches, we used local Bayesian multitrait models to estimate the genetic covariance between a marker for chronic kidney disease (estimated glomerular filtration rate) and serum urate in specific genomic regions. We identified 134 overlapping linkage disequilibrium windows with statistically significant covariance estimates, 49 of which had positive directionalities, and 85 negative directionalities, the latter being consistent with that of the overall genetic covariance. The 134 significant windows condensed to 64 genetically distinct shared loci which validate 17 previously identified shared loci with consistent directionality and revealed 22 novel pleiotropic genes. Finally, to examine potential biological mechanisms for these shared loci, we have identified a subset of the genomic windows that are associated with gene expression using colocalization analyses. The regions identified by our local Bayesian multitrait model approach may help explain the association between chronic kidney disease and hyperuricemia.

## Introduction

Chronic kidney disease (CKD) carries significant global health and economic burden (Hill *et al.* 2016; Bikbov *et al.* 2020). CKD stages 3–5 manifest as decreased renal function and are defined by elevated serum creatinine (sCr) or estimated glomerular filtration rate (eGFR) <60 ml/min/1.73 m^2^. Hyperuricemia is defined by serum urate (sU) concentration >6.8 mg/dl and is contributed to by deteriorating renal function (Sun *et al.* 2018). Hyperuricemia has several comorbidities associated with it, including CKD and gout (Clarson *et al.* 2015; Sun *et al.* 2018; Singh *et al.* 2019). Among people with hyperuricemia, there is a higher prevalence of CKD, and among patients with CKD, sU concentrations are higher (Zhu *et al.* 2012; Jing *et al.* 2018).

Genome-wide analyses have demonstrated that the association observed between eGFR and sU has a genetic basis. Tin *et al.* (2019) carried out a large-sample trans-ethnic genome-wide association study (GWAS) of sU and, through cross-trait linkage disequilibrium (LD) score regression, obtained an estimate of overall genetic correlation between eGFR and sU of −0.26 (SE of 0.04). This was one of the largest negative correlations with sU out of 748 traits analyzed (Tin *et al.* 2019). Reynolds *et al.*, using 2 large family-based datasets and Bayesian whole-genome regressions, obtained global genetic correlations between sCr (which has a direct inverse relationship to eGFR, hence the directionality difference between the estimates) and sU of 0.20 [95% credibility region (CR): 0.07, 0.33] in one dataset and 0.25 (95% CR: 0.07, 0.41) in the other (Reynolds *et al.* 2021). While these estimates contribute to dissecting biological causes of the observed comorbidities, the shared pleiotropic genomic regions and underlying biological mechanisms are only reliably discovered by estimating local genetic covariances (Shi *et al.* 2017).

GWAS of sU and eGFR have identified numerous loci associated with each phenotype separately. A recent study comparing large GWAS of these traits identified 36 shared loci (Leask *et al.* 2020). However, the GWAS methods used to detect the shared signals are based on the marginal association of individual single-nucleotide polymorphisms (SNPs) with phenotypes, thus not accounting for LD between SNPs. Our method improves over postanalysis of GWAS summary statistics by estimating neighboring SNP effects concomitantly. Incorporating local LD to estimate genetic effects in a tightly segregating chromosomal segment has been previously suggested to account for the correlation between SNPs (Vilhjálmsson *et al.* 2015; Fernando *et al.* 2017; Funkhouser *et al.* 2020). Additionally, our methodology implements a multitrait model so we obtain direct genetic covariance estimates.

In this study, we aimed to characterize the common genetic basis for CKD (eGFR) and hyperuricemia (sU levels) by identifying pleiotropic genomic regions. To achieve this goal, we identified the local regions contributing to genetic variances and covariances across the whole genome (Funkhouser *et al.* 2020). We used Bayesian multitrait models to estimate the genetic (co)variances. SNP effects were estimated in large DNA regions and genetic variances and covariances were calculated from the posterior means per LD window. We identified 64 unique local genetic regions with significant local genetic covariance, including previously implicated and novel shared loci.

## Materials and methods

### Participants

This study was based on 333,542 Caucasian participants from the UK Biobank. Participants missing sU or sCr for both of their 2 visits were excluded from the analysis. We excluded close relatives with relatedness ≥0.1, estimated using the R package BGData (Grueneberg and de los Campos 2019) (see details in the Supplementary Methods).

### Genotypes and phenotypes

The UK Biobank used the custom UK Biobank Axiom Array by Affymetrix to genotype study participants (Affymetrix 2021). Quality control involved removing SNPs that had a minor allele frequency <1% or a missing call rate >5%, resulting in 607,490 autosomal chromosomes (1–22) SNPs (Kim *et al.* 2017).

Serum urate and sCr data were obtained from the first visit. For the small number of participants (0.28%) that did not have phenotype data of interest collected at the first visit, we retrieved data from the second visit. sCr was used to define eGFR and details on this can be found in the Supplementary Methods. For both eGFR and sU, we took a *log* transformation to normalize their distributions and preadjusted by age, sex, and the first 5 SNP-derived principal components using ordinary least squares.

### Local Bayesian multitrait models

We estimated local (co)variances by fitting Bayesian models to chromosomal segments with a nonoverlapping core of 1,000 contiguous SNPs (between 3 and 4 Mbp depending on the region). We included 2 overlapping flanking regions each consisting of 250 SNPs to each side of the core. The SNPs in the flanking regions were included to account for the effects of SNPs that were outside of the core region but possibly in LD with SNPs in the core segment. Whole-genome regressions have been used to fit several markers concomitantly [e.g. Vazquez *et al.* (2012)]. However, biobank data impose computational restrictions due to its large dimensions. In the context of a single trait, local Bayesian conditional regressions have been employed to deal with the computational burden (Funkhouser *et al.* 2020). In their study, the authors indagated sex differences in genetic effects in single-trait models. Here, we utilized the idea of conditional regressions in large chunks of DNA with flanking regions in the context of a multitrait Bayesian model. This provides posterior estimates of variances and covariances between traits to find pleiotropic regions. The linear model used had the form Y= $1\mu^{\prime }$+ Xβ + E, where ***Y***_*n*__×2_ is a matrix containing the preadjusted phenotypes, μ_2__×__1_ is a vector of trait-specific intercepts, $X_{n\times 1,500}$ is an SNP-genotype matrix (1,000 core SNPs plus 250 flanking SNPs to each side), β_1__,__500__×__2_ is a matrix of SNP effects, and $E_{n\times 2}$ is a matrix of error terms. The error terms were assumed to be IID multivariate normal with a mean of zero and covariance $\mathrm{Var}\left(\varepsilon_{i}\right)$=R_2__×__2_, where $\varepsilon_{i}$ is the *i*th row of E. We used IID priors with a point of mass at zero and a bivariate Gaussian slab with a mean of zero and (co)variance matrix Σ_2__×__2_. The extent of shrinkage and variable selection was influenced by 3 groups of parameters: R, Σ, and the prior proportion of nonzero effects, π. For a 2-trait model, π={π_1_, π_2_} and represents the prior probability of nonzero effects for traits 1 and 2 (sU and eGFR), respectively. We treated the {R, Σ, π} parameters as unknown and we assigned Inverse-Wishart priors for the (co)variance matrices and Beta priors for the prior probability of nonzero effects.

We used the multitrait function from the BGLR R package available in the R CRAN (Pérez and de los Campos 2014) to generate 5,000 samples from the posterior distribution for each chromosomal segment. We filtered the samples of the SNP effects collected using a burn-in of 250 SNPs and a thinning interval of 10, thus retaining 475 samples for further inference.

### Defining local LD-based windows

After we obtained the model estimates, for each core segment SNP we defined an LD window that contained correlated, neighboring SNPs with an overlapping sliding technique (Fernando *et al.* 2017; Funkhouser *et al.* 2020). Within each LD window, we collected the corresponding estimated effects and computed (co)variance estimates (described below). For each seed SNP *x_ij_* (*i *=* *1,…,*n* individuals and *j *=* *1,…,*p* core segment SNPs) coming from the core segment of SNPs, we sequentially identified SNPs in both directions (x_ij*_) surrounding the seed SNP and included them in window *j* if Corr(*x_ij_*, x_ij*_) ≥ 0.1. In a simplified example, if SNP *x_ij_* had an adequate pairwise correlation with 2 SNPs to the left, and 1 SNP to the right, the window for that SNP would be defined as the set of SNPs: {*x_ij_*_−__2_, *x_ij_*_−__1_, *x_ij_*, *x_ij_*_+1_}. That is, Corr(*x_ij_*, *x_ij_*_−__1_) ≥ 0.1 and Corr(*x_ij_*, *x_ij_*_−__2_) ≥ 0.1 and Corr(*x_ij_*, *x_ij_*_+1_) ≥ 0.1. Our definition of an LD sliding window also involved an allowance for 1 SNP in the sequential process to not meet this correlation criterion, to allow for a brief loss of LD or minor mapping errors, and the SNP was still included in the LD window. In the previous example, if Corr(*x_ij_*, *x_ij_*_−__1_) < 0.1, and Corr(*x_ij_*, *x_ij_*_−__2_) ≥ 0.1, then the set would still include both *x_ij_*_−__2_ and *x_ij_*_−__1_. The LD window ends when 2 SNPs sequentially did not meet the criteria described above. The LD windows could include flanking buffer SNPs, but buffer SNPs were never used to define an LD window.

### Local (co)variances

For each LD window, we computed the local variances for traits 1 and 2 and the local and covariances using $V_{w1s}=\mathrm{Var}\left(X_{w}\beta_{w1s}\right)$, $V_{w2s}=\mathrm{Var}\left(X_{w}\beta_{w2s}\right)$, and $\mathrm{Co}v_{ws}=\mathrm{Cov}\left(X_{w}\beta_{w1s},X_{w}\beta_{w2s}\right)$. Here, $X_{w}$ is the matrix containing the genotypes of the SNPs in the *w*th window and $\beta_{w1s}$ and $\beta_{w2s}$ are the samples of effects of those SNPs for traits 1 and 2 collected at the *s*th iteration of the sampler. This generated samples from the posterior distribution of the local (co)variances, which we used to produce posterior mean estimates (by averaging across the samples from the posterior distribution), estimate posterior SDs, and obtain 95% posterior CRs. As discussed in Lehermeier *et al.* (2017), this approach accounts for the contribution of local LD to genetic (co)variances and, by averaging over samples from the posterior distribution, for uncertainty about SNP effects.

### Gene expression/eQTL analysis

A colocalization analysis was performed between GWAS significant markers for sU and sCr and the publicly available eQTL data from Genotype Tissue Expression (GTEx) V8 (Giambartolomei *et al.* 2014). The R package COLOC was used, which implements a Bayesian test that analyses a single genomic region and identifies LD patterns in that locus using SNP summary statistics and the associated minor allele frequencies. The lead variant for both sCr and sU was used at each significant covariance window with a surrounding 500 kb buffer in the GTEx database. The contextualizing developmental SNPs using 3D Information algorithm (Fadason *et al.* 2018; *Genome3d/Codes3d-V2* [2019] 2021) was modified to identify long-distance regulatory relationships for the lead sU and sCr variants at each significant covariance window within a 500-kb region. eQTL data for variants ±500 kb of the lead variant were also extracted from GTEx and then COLOC was used to assess if the significant *cis*- and *trans*-eQTL identified were colocalized with sCr and sU signals. An eQTL was determined to be colocalized if the COLOC H4 [posterior probability of colocalization (PPC)] was at least 0.5 for both traits and at least 0.8 for one of the 2 traits, according to Giambartolomei *et al.* (2014).

### Validation

We performed a validation analysis with the related Caucasian UK Biobank cohort, consisting of 57,370 subjects not missing sU or eGFR phenotypes. The genotyping array used for this cohort is the same as that used for the discovery analysis cohort. The validation analysis repeated the estimation procedures described above and the sliding LD windows used were identical to those used in the discovery set.

## Results

This study was based on 333,542 distantly related white participants, of whom 53.7% were female with an average age of 56.9 ± 8.0 years old. The average sCr level was 0.8 ± 0.2 mg/dl (the average ± SE), average eGFR was 144.2 ± 56.0 ml/min/1.73 m^2^, and the average sU level was 5.2 ± 1.3 mg/dl. Two (2.0) percent of the individuals had an ICD10 diagnosis or self-diagnosis of gout, 12.4% had hyperuricemia, 0.5% had CKD, and 0.3% had hyperuricemia and CKD.

**Fig. 1.**
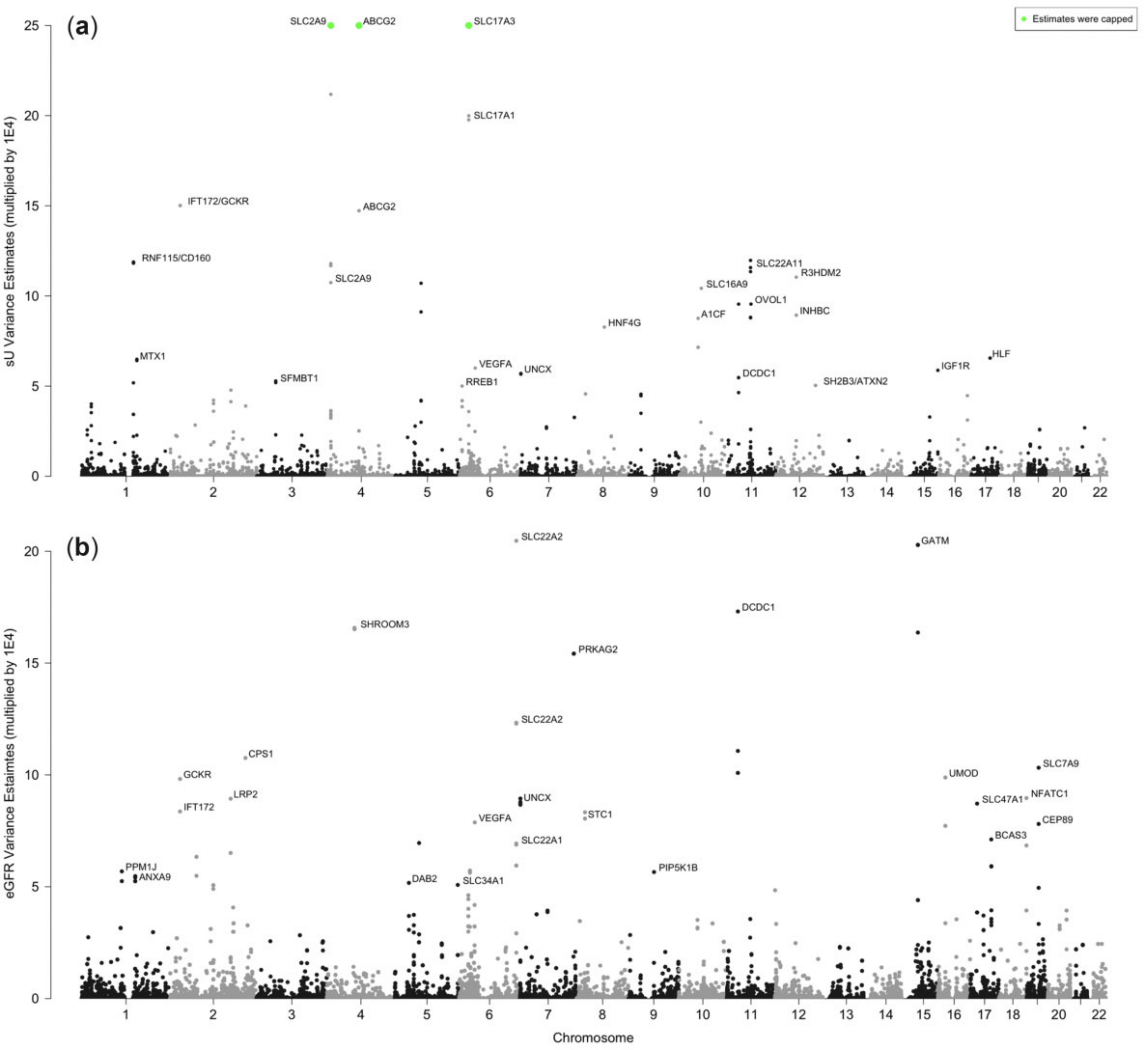
The variance estimates of overlapping LD windows. a) Variance estimates multiplied by 1E4 for sU concentrations and (b) for eGFR.

**Fig. 2.**
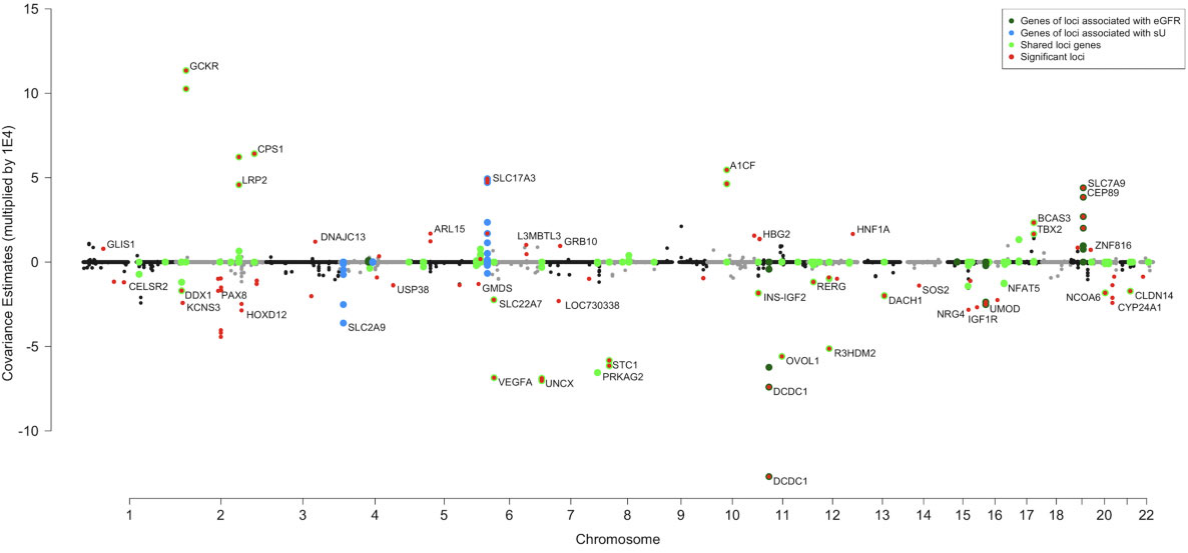
The covariance estimates of overlapping LD windows. Windows are selectively annotated with the gene name of the mid-point SNP of that window. Windows that contained SNPs in loci associated with known eGFR genes are highlighted in dark green, windows that contained SNPs in genes associated with sU are highlighted in blue, and windows that contained SNPs in genes associated with both sU and eGFR [from comparing GWAS, Leask *et al.* (2020)] are highlighted in bright green. Windows significant for genetic covariance are highlighted in red. The covariance estimates were multiplied by 1E4.

We analyzed the markers (sU and eGFR) using a sequence of Bayesian multitrait models where the markers were regressed on contiguous SNPs in a large chromosomal segment (core) plus overlapping flanking buffers. We collected the samples from the posterior distribution of effects for each core segment and used these samples to estimate the local variances for each marker (Fig. 1) and the local covariances between the markers (Fig. 2). The (co)variances were estimated within 511,828 overlapping LD windows (small, nonindependent contiguous chromosomal regions).

We found 134 LD windows with covariance estimates that had a 95% CR excluding zero (Fig. 2; Supplementary Table 1). The number of SNPs in the significant LD windows ranged from 1 to 56, and the median SNPs per window was 6.0 (22 kb on average, excluding 12 single-SNP windows). Interestingly, although the global correlation between sU and eGFR is negative (Tin *et al.* 2019; Reynolds *et al.* 2021), 49 of the 134 significant windows showed positive genetic covariance directionality, and the remaining 85 were negative.

**Fig. 3.**
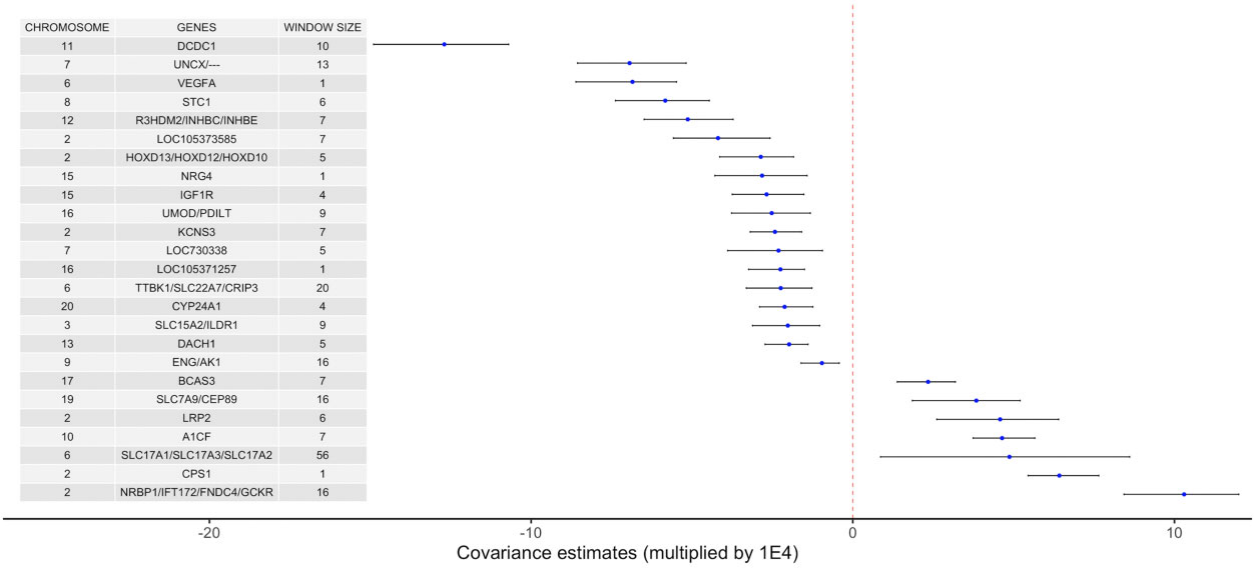
The top 25 shared loci and their covariance estimates with corresponding 95% CRs. The top 25 distinct loci from LD genomic regions with CRs not including zero. The window size indicates the number of SNPs in each window. The covariance estimates and CRs were multiplied by 1E4.

The 134 significant LD windows often included the same variants and mapped to identical GWAS loci, so we collapsed the 134 windows to 64 unique loci that possessed genetic covariance signal between eGFR and sU (Supplementary Table 2 and Supplementary Methods). The top 25 distinct loci implicated by the significant windows in terms of covariance magnitude are listed in Table 1. A graphical representation of the top significant loci is presented in Fig. 3.

**Table 1.** The top 25 magnitude genomic windows significant for covariance between sU and eGFR with their chromosome, annotated gene name, number of SNPs and first and last SNP names, estimated covariance [95% CR], and colocalized genes.

Chromosome	Annotated gene name	Number of SNPS in the window and first to last SNP	Estimated covariance [95% CR]^a^	Colocalized genes
2	*CPS1*	1	6.42	
		rs1047891	[5.45, 7.65]	
2	*LRP2*	6	4.58	
		rs41268683–rs2075252	[2.61, 6.4]	
2	*NRBP1/IFT172/FNDC4/GCKR*	16	10.3	*NRBP1*
		Affx-19857019–rs1260333	[8.43, 12]	
6	*SLC17A1/SLC17A3/SLC17A2*	56	4.87	
		rs1165196–rs9467632	[.863, 8.61]	
10	*A1CF*	7	4.64	*A1CF*
		rs12413118–rs61856594	[3.74, 5.66]	
17	*BCAS3*	7	2.34	*CRHBP*, *SH3GL2*
		rs9904048–rs9895661	[1.38, 3.19]	
19	*SLC7A9/CEP89*	16	3.84	*SLC7A9*, *CLDND2*
		rs78676942–rs11668957	[1.85, 5.2]	
2	*LOC105373585*	7	−4.19	
		rs11122800–rs35932591	[−5.58, −2.57]	
2	*HOXD13/HOXD12/HOXD10*	5	−2.86	
		rs847153–rs711818	[−4.14, −1.84]	
2	*KCNS3*	7	−2.42	
		rs9789415–rs11688124	[−3.19, −1.59]	
3	*SLC15A2/ILDR1*	9	−2.02	*SLC15A2*, *CD86*
		rs2049330–rs6438689	[−3.12, −1.03]	
6	*VEGFA*	1	−6.85	*SETD1A*
		rs881858	[−8.61, −5.48]	
6	*TTBK1/SLC22A7/CRIP3*	20	−2.24	*SETD1A*
		rs2651206–rs2242416	[−3.31, −1.27]	
7	*UNCX*	13	−6.94	*PALM2*, *PSMD11*
		rs6950388–rs1880301	[−8.56, −5.18]	
7	*LOC730338*	5	−2.31	
		rs700752–rs12537178	[−3.89, −9.44]	
8	*STC1*	6	−5.83	*RP11-38H17.1*
		rs62502212–rs1705690	[−7.38, −4.46]	
11	*OVOL1*	7	−5.59	*PCNX3*, *MAP3K11*, *SCYL1*, *RP-11-770G2.2*, *OVOL1*, *KRT8P26*
		rs4014195–rs36008241	[−8.13, −3.29]	
11	*DCDC1*	10	−12.7	
		rs963837–rs10767873	[−14.9, −10.7]	
12	*R3HDM2/INHBC/INHBE*	7	−5.13	*KMT2A*, *R3HDM2*, *SFXN5*
		rs73115999–rs507562	[−6.49, −3.72]	
13	*DACH1*	5	−1.98	
		rs7981995–rs626277	[−2.73, −1.39]	
15	*NRG4*	1	−2.82	*MAN2C1*, *PARD3*
		rs8024155	[−4.29, −1.42]	
15	*IGF1R*	4	−2.68	*IGF1R, NRCAM, TRAPPC10*
		rs907808–rs12437561	[−3.75, −1.52]	
16	*UMOD/PDILT*	9	−2.52	*ACSM1*, *DNAH3*
		rs1123670–rs12917707	[−3.77, −1.32]	
16	*LOC105371257*	1	−2.25	
		rs12927956	[−3.24, −1.5]	
20	*CYP24A1*	4	−2.12	
		rs4809954–rs2616278	[−2.9, −1.24]	

a Estimates and CRs were multiplied by 1E4 for readability.

### Gene expression/eQTL analysis

We used COLOC (Giambartolomei *et al.* 2014) and expression data from the GTEx project (v8) (Carithers and Moore 2015) to identify candidate causal genes at significant local genetic covariance windows between sU and eGFR. Twenty-six of the 64 distinct significant shared loci (41.6%) were shown to modify the expression of candidate causal genes colocalized with the covariance signals (Supplementary Table 3). Of note are *TRIM6* and *L3MBTL3* in *cis*, which are genes that have a significant covariance signal and a colocalized eQTL that is expressed in the kidney.

### Validation

In the related white UK Biobank validation cohort 12 LD windows were significant for genetic covariance between sU and eGFR (Supplementary Table 1). All of the 12 significant windows were also significant in the main analysis with consistent directionality. The 12 windows condensed to 5 distinct loci (Supplementary Table 2), meaning 5 out the 64 significant distinct loci from the main analysis were also significant in this validation. The sample size of the related cohort is 82.8% smaller (*n *=* *57,370) than the unrelated cohort used in the discovery set (*n *=* *333,542), so our validation analysis was comparatively underpowered to the main analysis.

## Discussion

The goal of this study was to infer the shared genetic architecture of sU (causal for gout), and eGFR (a marker for CKD). Our results highlight genes that may be involved in the observed relationship between the traits. In this study, we estimated local genetic (co)variances between sU and eGFR and identified regions with pleiotropy. This study was based on the large-scale UK Biobank and formal statistical inference from local Bayesian multitrait models. Our results demonstrated that genetic covariance between eGFR and sU was widespread across the genome. Our method identified 64 distinct LD windows with shared genetic effects between eGFR and sU, the majority of which had negative genetic covariance estimates. We identified 22 distinct novel shared loci, to our knowledge, with significant local genetic covariance for sU and eGFR, including *MMP11*/*SMARCB1*, *ADH1B*, *MIP*/*GLS2*, *ENG*/*AK1*, *EPB41L5*, *KIAA1199*, *CELSR2*, *SOS2*, *KCNS3*, *TET2*, *SMLR1*/*EPB41L2*, *GLIS1*, *KIAA1683*/*JUND*, and *METTL10*/*FAM175B*. Furthermore, 14 distinct loci identified were previously only known to be associated with only one of the 2 traits, demonstrating that the set of loci contributing to both traits is substantially larger than previously thought. These loci are partially responsible for the comorbidity between hyperuricemia/gout and CKD.

One advantage of the local method that we present here is that it facilitates the identification of genomic windows with opposite signs to the overall negative genetic correlation between eGFR and sU. Out of the significant shared loci, about two-thirds showed negative local genetic covariance estimates. This is consistent with the overall genetic covariance directionality (Tin *et al.* 2019; Reynolds *et al.* 2021), indicating that they either contribute to worsening kidney function (decreasing eGFR or increasing sCr) and increasing sU, or vice versa. Interestingly, there were 21 distinct significant shared loci with positive local genetic covariance estimates (about one-third). Positive covariance indicates that the genomic region either contributes to increasing sU and improved kidney function or decreasing sU and worsening kidney function. Two of the loci with a significant positive signal, *GCKR* and *CPS1*, are mainly expressed in the liver and one, *LRP2*, is mainly expressed in the kidney (Carithers and Moore 2015). One novel shared locus identified in this study consisted of the genes *SLC17A1*, *SLC17A3*, and *SLC17A2*. This large window in chromosome 6 (56 SNPs, Table 1) had a strong, positive significant covariance signal and *SLC17A1* and *SLC17A3* are urate transporters both linked to gout (Reimer 2013). The opposite signs of locus-specific genetic covariances are indicative of distinct physiological processes governing the phenotypic expression of urate and eGFR. The loci with positive covariance in particular are excellent candidates for discovering functional mechanisms that simultaneously increase sU and improve kidney function.

Urate transporters *SLC2A9* and *ABCG2* have the largest GWAS effect sizes for sU, accounting for 4–5% of the variance in sU (Yang *et al.* 2010; Hughes *et al.* 2014; Johnson *et al.* 2018; Major *et al.* 2018; Tin *et al.* 2019). However, no windows in *SLC2A9* or *ABCG2* had a 95% CR for local genetic covariance that did not include zero. Our results demonstrate that windows in both *SLC2A9* and *ABCG2* loci are associated with just sU levels but are not pleiotropic regions for sU and eGFR. A similar phenomenon is observed with the eGFR gene *SHROOM3*. That is, none of the windows containing SNPs in *SHROOM3* were significant for local genetic covariance. This exemplifies that the loci driving the genetic correlation between these 2 traits are not necessarily the leading GWAS hits.

Previous research investigating pleiotropic genetic loci between sU and eGFR has implicated loci as shared if signals of association obtained from marginal single-marker regressions (e.g. GWAS) for both traits are colocalized (Leask *et al.* 2020). Leask *et al.* (2020) recently compared overlapping loci between 2 large GWAS, one of sU and the other kidney function (Wuttke and Köttgen 2016; Tin *et al.* 2019), and found 36 independent colocalized loci. Our results validate 20 of these 36 loci, and all but 3 loci (*DACH1*, *CPS1*, and *INS-IGF2*) had covariance directionality that matched the directionality of effects found by Leask *et al.* (2020).

Our covariance approach may have direct implications for assessing causal relationships between exposures using Mendelian randomization (MR). Pleiotropic genetic variants violate assumptions of univariate MR, however, they are useful in multivariable MR that can simultaneously assess the causal effects of multiple risk factors on an outcome (Burgess and Thompson 2015). For example, genetic variants from *SLC2A9* and *ABCG2* may be valid instrumental variables to use in MR to test for a causal effect of sU on CKD, however, the loci listed in Supplementary Table 1 would not. In fact, *SLC22A11* has previously been identified as a pleiotropic variant that may improve kidney function through its activity in raising urate levels (Hughes *et al.* 2014). MR has previously been used to show that sU is not causal of CKD (Jordan *et al.* 2019), however, Jordan *et al.* noted significant pleiotropy in the genetic variants used in their study, which they attempted to counter using MR techniques robust to pleiotropy. Of the 26 SNPs used by Jordan *et al.*, rs1260326 (*GCKR*) and rs17050272 (*LINC01101*) were identified by us as shared, and rs1165151 and rs3741414 were located within one of our significant pleiotropic regions but were not in our genotyping platform.

Our eQTL analysis of the windows significant for local genetic covariance uncovered numerous genes of interest, such as *SLC7A9*, which encodes a solute transporter largely expressed in the small intestine, *A1CF*, which encodes a protein involved in apolipoprotein B synthesis in the liver, and *TRIM6*, which encodes an E3 ubiquitin ligase involved in interferon gamma signaling and innate immune response with high expression levels in the kidney (Carithers and Moore 2015). The genes uncovered from the eQTL analysis will be particularly interesting for future study, as they will likely aid our understanding of the relationship between kidney function and sU.

Through our approach of obtaining local genetic (co)variance estimates from Bayesian multitrait models in very large datasets, we have uncovered 22 novel shared genetic regions for sU and eGFR. The approach presented in this paper was applied in the context of sU and eGFR, but it could be applied to any pair of traits. While our discovery set sample size is excellent, we lack a dataset of a similar size for the validation. Some regions were validated but not all.

The local shared genomic regions we have uncovered in this study can provide insight into the relationship between hyperuricemia/gout and CKD, elucidating the biological mechanisms underlying the traits. This will help further understanding of the genetic basis of hyperuricemia/gout and CKD.

## Data availability

All data used are secondary and are held in public repositories. This study utilized deidentified data from the UK Biobank where genotype and phenotype data are available to researchers upon registration. The protocol and consent were approved by the UK Biobank’s Research Ethics Committee and were conducted under the application number “15326.” For eQTL analysis, *cis*- and *trans*-eQTL data were downloaded from the GTEx V8 portal (Carithers and Moore 2015).

Supplemental material is available at *G3* online.

## Supplementary Material

jkac158_Supplementary_Methods

jkac158_Supplementary_Table_1_2

jkac158_Supplementary_Table_3

## Notes

UK Biobank: https://www.ukbiobank.ac.uk/

## References

[jkac158-B1] Affymetrix. Genetic Data: detailed Genetic Data on Half a Million People. 2021. [accessed 2021 Feb 26]. http://www.ukbiobank.ac.uk/scientists-3/uk-biobank-axiom-array/.

[jkac158-B2] Bikbov B , Purcell CA, Levey AS, Smith M, Abdoli A, Abebe M, Adebayo OM, Afarideh M, Agarwal SK, Agudelo-Botero M, et al Global, regional, and national burden of chronic kidney disease, 1990–2017: a systematic analysis for the global burden of disease study 2017. Lancet. 2020;395(10225):709–733. doi:10.1016/S0140-6736(20)30045-3. 32061315 PMC7049905

[jkac158-B3] Burgess S , Thompson SG. Multivariable Mendelian randomization: the use of pleiotropic genetic variants to estimate causal effects. Am J Epidemiol. 2015;181(4):251–260. doi:10.1093/aje/kwu283. 25632051 PMC4325677

[jkac158-B4] Carithers LJ , Moore HM. The genotype-tissue expression (GTEx) project. Biopreserv Biobank. 2015;13(5):307–308. doi:10.1089/bio.2015.29031.hmm. 26484569 PMC4692118

[jkac158-B5] Clarson LE , Hider SL, Belcher J, Heneghan C, Roddy E, Mallen CD. Increased risk of vascular disease associated with gout: a retrospective, matched cohort study in the UK clinical practice research datalink. Ann Rheum Dis. 2015;74(4):642–647. doi:10.1136/annrheumdis-2014-205252. 25165032 PMC4392302

[jkac158-B7] Fadason T , Schierding W, Lumley T, O'Sullivan JM. Chromatin interactions and expression quantitative trait loci reveal genetic drivers of multimorbidities. Nat Commun. 2018;9(1):5198. doi:10.1038/s41467-018-07692-y. 30518762 PMC6281603

[jkac158-B8] Fernando R , Toosi A, Wolc A, Garrick D, Dekkers J. Application of whole-genome prediction methods for genome-wide association studies: a Bayesian approach. J Agric Biol Environ Stat. 2017;22(2):172–193. doi:10.1007/s13253-017-0277-6.

[jkac158-B9] Funkhouser SA , Vazquez AI, Steibel JP, Ernst CW, de los Campos G. Deciphering sex-specific genetic architectures using local Bayesian regressions. Genetics. 2020;215(1):231–241. doi:10.1534/genetics.120.303120. 32198180 PMC7198271

[jkac158-B10] Genome3d/Codes3d-*V2* [2019]. Python. Genome3d; 2019. [accessed: 2021 Feb 26]. https://github.com/Genome3d/codes3d-v2.

[jkac158-B11] Giambartolomei C , Vukcevic D, Schadt EE, Franke L, Hingorani AD, Wallace C, Plagnol V. Bayesian test for colocalisation between pairs of genetic association studies using summary statistics. PLoS Genet. 2014;10(5):e1004383. doi:10.1371/journal.pgen.1004383. 24830394 PMC4022491

[jkac158-B12] Grueneberg A , de los Campos G. BGData—a suite of R packages for genomic analysis with big data. 2019;9(5):1377–1383. doi:10.1534/g3.119.400018. PMC6505159 30894453

[jkac158-B13] Hill NR , Fatoba ST, Oke JL, Hirst JA, O’Callaghan CA, Lasserson DS, Hobbs FDR. Global prevalence of chronic kidney disease—a systematic review and meta-analysis. PLoS One. 2016;11(7):e0158765. doi:10.1371/journal.pone.0158765. 27383068 PMC4934905

[jkac158-B14] Hughes K , Flynn T, de Zoysa J, Dalbeth N, Merriman TR. Mendelian randomization analysis associates increased serum urate, due to genetic variation in uric acid transporters, with improved renal function. Kidney Int. 2014;85(2):344–351. doi:10.1038/ki.2013.353. 24048376 PMC5665684

[jkac158-B15] Jing J , Ekici AB, Sitter T, Eckardt K-U, Schaeffner E, Li Y, Kronenberg F, Köttgen A, Schultheiss UT. Genetics of serum urate concentrations and gout in a high-risk population, patients with chronic kidney disease. Sci Rep. 2018;8(1):13184. doi:10.1038/s41598-018-31282-z. 30181573 PMC6123425

[jkac158-B16] Johnson RJ , Bakris GL, Borghi C, Chonchol MB, Feldman D, Lanaspa MA, Merriman TR, Moe OW, Mount DB, Sanchez Lozada LG, et al Hyperuricemia, acute and chronic kidney disease, hypertension, and cardiovascular disease: report of a scientific workshop organized by the National Kidney Foundation. Am J Kidney Dis. 2018;71(6):851–865. doi:10.1053/j.ajkd.2017.12.009. 29496260 PMC7286363

[jkac158-B17] Jordan DM , Choi HK, Verbanck M, Topless R, Won H-H, Nadkarni G, Merriman TR, Do R. No causal effects of serum urate levels on the risk of chronic kidney disease: a Mendelian randomization study. PLoS Med. 2019;16(1):e1002725. doi:10.1371/journal.pmed.1002725. 30645594 PMC6333326

[jkac158-B18] Kim H , Grueneberg A, Vazquez AI, Hsu S, de Los Campos G. Will big data close the missing heritability gap? Genetics. 2017;207(3):1135–1145. doi:10.1534/genetics.117.300271. 28893854 PMC5676235

[jkac158-B19] Leask MP , Sumpter NA, Lupi AS, Vazquez AI, Reynolds RJ, Mount DB, Merriman TR. The shared genetic basis of hyperuricemia, gout, and kidney function. Sem Nephrol. 2020;40(6):586–599. doi:10.1016/j.semnephrol.2020.12.002. 33678313

[jkac158-B20] Lehermeier C , de Los Campos G, Wimmer V, Schön C-C. Genomic variance estimates: with or without disequilibrium covariances? J Anim Breed Genet. 2017;134(3):232–241. doi:10.1111/jbg.12268. 28508483

[jkac158-B21] Levey AS , Stevens LA, Schmid CH, Zhang Y, Castro AF, Feldman HI, Kusek JW, Eggers P, Van Lente F, Greene T, et al; for the CKD-EPI (Chronic Kidney Disease Epidemiology Collaboration. A new equation to estimate glomerular filtration rate. Ann Intern Med. 2009;150(9):604–612. doi:10.7326/0003-4819-150-9-200905050-00006. 19414839 PMC2763564

[jkac158-B22] Major TJ , Dalbeth N, Stahl EA, Merriman TR. An update on the genetics of hyperuricaemia and gout. Nat Rev Rheumatol. 2018;14(6):341–353. doi:10.1038/s41584-018-0004-x. 29740155

[jkac158-B23] Pérez P , de los Campos G. Genome-wide regression and prediction with the BGLR statistical package. Genetics. 2014;198(2):483–495. doi:10.1534/genetics.114.164442. 25009151 PMC4196607

[jkac158-B24] Reimer RJ. *SLC17*: a functionally diverse family of organic anion transporters. Mol Aspects Med. 2013;34(2–3):350–359. doi:10.1016/j.mam.2012.05.004. 23506876 PMC3927456

[jkac158-B25] Reynolds RJ , Irvin MR, Bridges SL, Kim H, Merriman TR, Arnett DK, Singh JA, Sumpter NA, Lupi AS, Vazquez AI. Genetic correlations between traits associated with hyperuricemia, gout, and comorbidities. Eur J Hum Genet. 2021;29(9):1438–1445. doi:10.1038/s41431-021-00830-z. 33637890 PMC8440599

[jkac158-B26] Shi H , Mancuso N, Spendlove S, Pasaniuc B. Local genetic correlation gives insights into the shared genetic architecture of complex traits. Am J Hum Genet. 2017;101(5):737–751. doi:10.1016/j.ajhg.2017.09.022. 29100087 PMC5673668

[jkac158-B27] Singh G , Lingala B, Mithal A. Gout and hyperuricaemia in the USA: prevalence and trends. Rheumatology (Oxford, England). 2019;58(12):2177–2180. doi:10.1093/rheumatology/kez196. 31168609

[jkac158-B28] Sun M , Vazquez AI, Reynolds RJ, Singh JA, Reeves M, Merriman TR, Gaffo AL, de Los Campos G. Untangling the complex relationships between incident gout risk, serum urate, and its comorbidities. Arthritis Res Therapy. 2018;20(1):90. doi:10.1186/s13075-018-1558-3. PMC5932762 29720278

[jkac158-B29] Tin A , Marten J, Halperin Kuhns VL, Li Y, Wuttke M, Kirsten H, Sieber KB, Qiu C, Gorski M, Yu Z, et al; V. A. Million Veteran Program. Target genes, variants, tissues and transcriptional pathways influencing human serum urate levels. Nat Genet. 2019;51(10):1459–1474. doi:10.1038/s41588-019-0504-x. 31578528 PMC6858555

[jkac158-B30] Vazquez AI , de los Campos G, Klimentidis YC, Rosa GJM, Gianola D, Yi N, Allison DB. A comprehensive genetic approach for improving prediction of skin cancer risk in humans. Genetics. 2012;192(4):1493–1502. doi:10.1534/genetics.112.141705. 23051645 PMC3512154

[jkac158-B31] Vilhjálmsson BJ , Yang J, Finucane HK, Gusev A, Lindström S, Ripke S, Genovese G, Loh P-R, Bhatia G, Do R, et al; Schizophrenia Working Group of the Psychiatric Genomics Consortium, Discovery, Biology, and Risk of Inherited Variants in Breast Cancer (DRIVE) Study. Modeling linkage disequilibrium increases accuracy of polygenic risk scores. Am J Hum Genet. 2015;97(4):576–592. doi:10.1016/j.ajhg.2015.09.001. 26430803 PMC4596916

[jkac158-B32] Wuttke M , Köttgen A. Insights into kidney diseases from genome-wide association studies. Nat Rev Nephrol. 2016;12(9):549–562. doi:10.1038/nrneph.2016.107. 27477491

[jkac158-B33] Yang Q , Köttgen A, Dehghan A, Smith AV, Glazer NL, Chen M-H, Chasman DI, Aspelund T, Eiriksdottir G, Harris TB, et al Multiple genetic loci influence serum urate levels and their relationship with gout and cardiovascular disease risk factors. Circ Cardiovasc Genet. 2010;3(6):523–530. doi:10.1161/CIRCGENETICS.109.934455. 20884846 PMC3371395

[jkac158-B34] Zhu Y , Pandya BJ, Choi HK. Comorbidities of gout and hyperuricemia in the US General Population: NHANES 2007–2008. Am J Med. 2012;125(7):679–687.e1. doi:10.1016/j.amjmed.2011.09.033. 22626509

