# Correction to: Local genetic covariance between serum urate and kidney function estimated with Bayesian multitrait models

**DOI:** 10.1093/g3journal/jkac259

**Published:** 2022-10-03

This is a correction to: Alexa S Lupi, Nicholas A Sumpter, Megan P Leask, Justin O’Sullivan, Tayaza Fadason, Gustavo de los Campos, Tony R Merriman, Richard J Reynolds, Ana I Vazquez, Local genetic covariance between serum urate and kidney function estimated with Bayesian multitrait models, *G3 Genes|Genomes|Genetics*, Volume 12, Issue 9, September 2022, jkac158, https://doi.org/10.1093/g3journal/jkac158

In the originally published version of this manuscript, a footnote to the author byline was missing:

“RJR and AIV share senior authorship on this work.”

This error has been corrected online.

