# Supplementary material for: Local genetic covariance between serum urate and kidney function estimated with Bayesian multitrait models
Source: G3 (Bethesda). 2022 Jul 25;12(9):jkac158. doi: 10.1093/g3journal/jkac158 (PMC9434310; doi:10.1093/g3journal/jkac158)
Supplement: jkac158_Supplementary_Methods [file jkac158_supplementary_methods.docx]

**Supplementary Methods for Lupi *et al.* 2022**

**Table of Contents**

***Supplementary Methods for Lupi et al. 2022***.................................................................................**1**

**Identification of distantly related samples**.............................................................................**1**

**Phenotypes**................................................................................................................................**1**

**Defining distinct loci**................................................................................................................**2**

**References**.................................................................................................................................**3**

**Identification of distantly related samples**

We used the R package BGData (Grueneberg and de los Campos 2019) to compute the expected proportion of allele sharing among UK Biobank individuals with the additive genomic relationship matrix ***G***, $\boldsymbol{G}=\frac{\boldsymbol{Z}\boldsymbol{Z}^{\boldsymbol{'}}}{tr(\boldsymbol{Z}\boldsymbol{Z}^{\boldsymbol{'}})/n}$ , where ***Z*** is a matrix of centered genotypes. That is, ***Z****_ij_* = *x_ij_* - 2*p_j_* where *x_ij_* is the number of copies of the reference allele at the *j*^th^ loci of the *i*^th^ individual and *p_j_* is the frequency of the reference allele of the *j*^th^ loci. In a homogeneous sample, *g_ij_* (where *i* ≠ *j*) can be considered as an estimate of the relatedness between subjects *i* and *j*. If *g_ij_* ≥ 0.1 they were excluded from the sample.

**Phenotypes**

eGFR is an indicator of renal function and was used to ascertain CKD. In this study, we defined eGFR using the abbreviated Modification of Diet in Renal Disease (MDRD) equation, which uses fewer variables than others yet performs just as well (Levey et al. 2009), with a modification to include a calibration factor to correct for the variability of sCr measures across laboratories and time (Coresh et al. 2002): *eGFR* = 186.3 × (*sCr* – 0.24) ^-1.154^ × *Age* ^-0.203^ × (0.742 if *Female*).

**Defining distinct loci**

We condensed our 134 significant windows to 64 distinct, non-overlapping regions. To determine which significant window would represent each region, we first checked if a window’s base pair position overlapped with that of a neighboring window. If the windows overlapped, we kept whichever window had the most SNPs. If the number of SNPs in the windows were equal, we kept the first of the two. This iterative process ended once there were no overlapping neighboring significant windows.

**Supplementary Methods References**

Coresh, Josef, Brad C. Astor, Geraldine McQuillan, John Kusek, Tom Greene, Frederick Van Lente, and Andrew S. Levey. 2002. “Calibration and Random Variation of the Serum Creatinine Assay as Critical Elements of Using Equations to Estimate Glomerular Filtration Rate.” *American Journal of Kidney Diseases* 39 (5): 920–29. https://doi.org/10.1053/ajkd.2002.32765.

Grueneberg, Alexander, and Gustavo de los Campos. 2019. “BGData - A Suite of R Packages for Genomic Analysis with Big Data.” *G3&amp;#58; Genes|Genomes|Genetics* 9 (5): 1377–83. https://doi.org/10.1534/g3.119.400018.

Levey, Andrew S., Lesley A. Stevens, Christopher H. Schmid, Yaping Lucy Zhang, Alejandro F. Castro, Harold I. Feldman, John W. Kusek, et al. 2009. “A New Equation to Estimate Glomerular Filtration Rate.” *Annals of Internal Medicine* 150 (9): 604–12. https://doi.org/10.7326/0003-4819-150-9-200905050-00006.
